# Assessing the Impact of Bedaquiline, Clofazimine, and Linezolid on Mycobacterial Genome Integrity

**DOI:** 10.3390/biom14111451

**Published:** 2024-11-15

**Authors:** Dániel Molnár, Éva Viola Surányi, Nikoletta Gálik, Judit Tóth, Rita Hirmondó

**Affiliations:** 1 Institute of Molecular Life Sciences, HUN-REN Research Centre for Natural Sciences, 1117 Budapest, Hungary; molnar.daniel@ttk.hu (D.M.);; 2 Doctoral School of Biology and Institute of Biology, ELTE Eötvös Loránd University, 1117 Budapest, Hungary

**Keywords:** drug resistance, second-line antituberculotics, mycobacteria, genome integrity, DNA repair

## Abstract

Tuberculosis (TB) presents significant medical challenges, largely due to the genetic diversity of *Mycobacterium tuberculosis*, which enhances the resilience and resistance of the pathogen to first-line treatments. In response to the global rise of drug-resistant TB, second-line antitubercular drugs like bedaquiline (BDQ), linezolid (LZD), and clofazimine (CFZ) have become critical treatment options. Understanding the molecular changes these drugs induce is essential for optimizing TB therapy. To contribute to this effort, we investigated their impact on genome maintenance and stability using *Mycobacterium smegmatis* as a model organism. Using mutation accumulation assays and whole-genome sequencing, we found that the second-line antibiotics did not significantly increase mutation rates, unlike the positive control UV treatment. However, upon BDQ treatment, we detected mutations in transporter proteins and transcription factors without any increase in the minimal inhibitory concentration. Additionally, BDQ and CFZ were found to alter DNA repair pathways and reduce cellular dNTP levels, particularly CFZ, which depleted dGTP, impacting DNA synthesis. CFZ also upregulated DNA repair enzymes, enhancing error-free repairs. Despite minimal mutagenic effects, both drugs displayed distinct impacts on cellular mechanisms, suggesting additional modes of action.

## 1. Introduction

The mycobacterial genus comprises some of the most ancient human pathogens including the causative agents of tuberculosis (TB) and leprosy. Other mycobacteria are the etiology of important diseases in humans and a wide range of animal species including domestic livestock and wildlife. The fact that *Mycobacteria* imposes a persistent global health and economic concern is mainly attributed to their unique cell wall [1] and lifestyle, leading to exclusive insensitivity to most antibiotics [2]. In addition, *Mycobacteria* demonstrate a tendency to develop resistance to the limited number of effective antimicrobial agents [3,4,5]. In 2022, the incidence of multidrug- or rifampicin-resistant TB (MDR/RR-TB) was estimated at 3.3% among new TB cases and 17% among those who had undergone prior treatment [6]. Despite these numbers showing a declining trend, resistance continues to pose a significant challenge to TB care [6], necessitating the understanding of resistance development and the corresponding adjustments of treatment regimens.

Second-line antituberculotics are medications used specifically for the treatment of drug-resistant tuberculosis (DR-TB). Commonly used second-line antituberculotics include the newly developed bedaquiline (BDQ) [7,8], and well-established antibiotics repurposed for TB treatment like linezolid (LZD) and clofazimine (CFZ) [9,10]. BDQ inhibits the mycobacterial ATP synthesis [11], and thus all ATP-dependent metabolic processes [12]. LZD is a protein synthesis inhibitor that binds to rRNA and uniquely blocks the initiation step of translation [13]. CFZ has been used in clinics for leprosy treatment since the 1950s in absence of the nowadays rigorous pharmacokinetic descriptive studies [14]. Based on what we know about the mechanism of action of CFZ, it has the potential to influence a diverse range of cellular processes. It was reported to bind nucleic acid polymers through its interaction with the guanine bases [15]. This mode of action would inhibit the template function of DNA, especially efficiently in the GC-rich genome of mycobacteria [15]. In addition, it has been demonstrated in *Mycobacterium smegmatis* (*M. smegmatis*) that CFZ induces the production of intracellular reactive oxygen species, suggesting that this could be its most effective mode of action [16]. CFZ also acts on membranes probably due to its extremely hydrophobic nature [17,18]. It can inhibit biofilm formation in both *M. tuberculosis* and *M. smegmatis* cultures [18]. CFZ demonstrated bactericidal effects against both actively dividing and slowly replicating bacilli, but it did not exhibit such activity against non-replicating organisms of either species [18]. This highlights the significance of its mode of action on nucleic acids. In summary, the mechanism of action of CFZ is not fully understood, and most of the downstream molecular effects of the observations have not yet been investigated.

Even though these are effective medications, resistance to second-line antituberculotics also occurs. Approximately 15% of MDR/RR TB progresses to an extensively drug-resistant (XDR) status, defined as resistance to rifampicin, fluoroquinolone, and at least one of BDQ or LZD [6]. Whether resistance development is linked to a potential increase in the mutagenesis rate induced by the stress the medication imposes on the organisms remains elusive. Consequently, in the selection of treatment, one unexplored but important consideration may be the potential mutator effect of these drugs on mycobacteria.

In this study, our aim was to assess how prolonged drug exposure affects the mutation rate and DNA repair system of mycobacteria, while also expanding our understanding of the mechanism of action of CFZ. Our selection of second-line antituberculosis drugs was guided by WHO recommendations for extended MDR-TB regimens (18 months), where we recognized the highest potential risk for mutation development under prolonged antibiotic pressure. For such extended treatments, the WHO advises a grouping of drugs that includes all three Group A agents (bedaquiline, linezolid, and levofloxacin or moxifloxacin), along with at least one Group B agent (such as clofazimine or cycloserine).

We omitted fluoroquinolones and cell wall biosynthesis inhibitors whose mechanisms of action are well documented. We employed the widely used laboratory model organism *M. smegmatis* [19], which shares genome metabolic pathways with medically significant mycobacterial species [20,21], making it a suitable model for studying fundamental molecular processes. We subjected mycobacteria to sublethal concentrations of BDQ, LZD, and CFZ, and examined the transcriptional changes in the DNA repair system, the cellular dNTP pools, and the long-term mutator effect by whole genome sequencing (WGS).

## *2.* Methods and Materials

### 2.1. Bacterial Strains, Media, Growth Conditions, and Viability Measurement

*M. smegmatis* mc^2^ 155 [22] strains were cultivated in Lemco broth (5 g/L Lab-Lemco, 5 g/L NaCl, 10 g/L Bacto peptone, 0.05% Tween-80) or on solid Lemco plates (6.25 g/L Lab-Lemco, 6.25 g/L NaCl, 12.5 g/L Bacto peptone, 18.75 g/L Bacto agar). The viability values presented in Table 1 are calculated based on the total colony forming units (CFUs) of each culture (liquid and agar plates). Liquid cultures were treated with specific concentrations of the applied drug for 8 h and CFU values were determined on antibiotic-free agar plates. In the case of agar plate optimization, CFU values of non-treated *M. smegmatis* liquid mid-exponential cultures were determined on stress-free or antibiotic-containing plates. All treatments were compared to non-treated controls. The optimization of treatments is shown in Figure S1.

### 2.2. Microscopy Analysis

For microscopic analysis, stress-treated and control cells (200-200 µL each) were retrieved before RNA or dNTP extraction and washed with PBS containing 0.1% Triton X-100. The cells were then fixed in 4% PFA dissolved in PBS for 30 min at 37 °C. Following fixation, cells were stained with 10 µg/mL DAPI for 30 min at 37 °C, then streaked onto microscopy slides covered with 0.1% low melting agarose (Sigma, St. Louis, MO, USA). Imaging was performed on a Leica DM IL LED (Leica, Wetzlar, Germany) microscope using phase-contrast and fluorescent modes. To quantify cell size and cell volume, ImageJ (1.54k) was used. The calculated cell metrics with sample sizes and raw microscopic images are available with the following DOI: 10.6084/m9.figshare.25218191.

### 2.3. Mutation Accumulation (MA) Experiments

Sixteen independent lines of the *M. smegmatis* mc^2^ 155 strain were initiated from a single colony for every treatment. To establish a single common ancestor, the ancestor cell colony underwent five streaking iterations from plate to plate before the initiation of treatments. Lemco agar medium was used for the MA line transfers. All MA lines were incubated at 37 °C. For the treatments, the applied antibiotic concentrations are outlined in Table 1. A single isolated colony from each MA line was transferred by streaking to a new plate every 3 days. The treatments lasted for 60 days, corresponding to approximately 230 cell divisions for each line. Generation times were calculated using the formula:

T_d_ = t/log_2_ (N_t_/N_0_)

where T_d_ is the generation time, t is the time interval between measurements, and N_t_ and N_0_ are the final and initial population sizes determined by CFU counting, respectively. Some mock treatments were extended to 120 days to ensure a presumably higher accumulation of mutational events without treatment. After completing the MA procedure, a single colony from each strain was transferred to a new plate without any treatment to facilitate growth for an additional 3 days. Subsequently, frozen stocks of all lineages were prepared in 20% glycerol and stored at −80 °C.

### 2.4. Assessment of Drug Tolerance Following MA Experiments

The development of tolerance to the applied treatment was assessed by measuring the minimal inhibitory concentration (MIC) of both the mock-treated and stressed MA strains. Three randomly chosen strains from frozen stocks from both the mock-treated and stress-treated groups were resuscitated on plates containing the same stress conditions as those used in the MA experiment. Liquid cultures were inoculated and diluted to an OD(600) of 0.001 in sterile, round-bottom 96-well plates (Sarstedt). The wells contained the specific drug in 2-fold serial dilution for both the stressed strains and control samples. Cells were grown at 37 °C without agitation. Plates were scanned and analyzed, and MIC values were determined based on the last well in which cell growth was observed.

### 2.5. DNA Extraction

At the end of the MA experiment, a single colony from all lineages was inoculated into 10 mL liquid culture. Mycobacteria were harvested as the cultures reached an optical density at 600 nm (OD600) of 0.8–1.0. Five to six cultures from individual lineages from the same treatment with similar estimated cell number (based on OD measurements) were pooled before genomic DNA isolation. For cell disruption, the cells were resuspended in 1 mL of 10 mM Tris, pH 7.5, and 0.1-mm glass beads were added to a final volume of 1.5 mL. Cell disruption was performed using a cell disruptor (Scientific Industries, SI-DD38 Digital Disruptor Genie Cell Disruptor, New York, NY, USA) in a cold room (at 4 °C). Following centrifugation for 10 min at 3220× *g*, at room temperature, DNA was extracted from the supernatant using phenol:chloroform:IAA (25:24:1) extraction, followed by isopropanol precipitation. The quality and quantity of the extracted DNA were assessed using UV photometry in a Nanodrop-2000 instrument (Thermo Scientific, Wilmington, DE, USA) and by agarose gel electrophoresis. 

### 2.6. DNA Library Preparation and Whole Genome Sequencing

Whole genome sequencing (WGS), including DNA library preparation, was conducted at Novogene Ltd., Beijing, China. Sequencing was executed on Illumina 1.9 instruments (Illumina Inc., San Diego, CA, USA) with 600-basepair (bp) fragments as 2 × 150 bp paired end sequencing. An average read depth of 267 was achieved across all samples.

### 2.7. WGS Analysis and Mutation Identification

Three parallel pooled samples were sequenced for every treatment, each containing five to six individually treated MA lineages, adding up to a subtotal of 16 individual lineages. The mock and UV radiation controls included in the analysis were identical with those used in a parallel study [23], as can be tracked in the deposited sequencing data and analysis scripts. The quality of the raw reads was analyzed using FastQC v.0.11.9. Adapters and low-quality bases (Phred score < 20) were trimmed using Trimmomatic v0.38 [24]. We mapped our paired end reads to the *M. smegmatis* mc^2^ 155 reference genome (GenBank accession number: NC_008596.1) by Bowtie2 v2.5.4 [25]. PCR duplicates were removed using Samblaster v0.1.26 [26]. We converted SAM files to BAM files and sorted them using SAMtools v1.20 [27]. Read groups were replaced by the Picard tool v2.23.3. Single nucleotide variations (SNVs), insertions, and deletions were called from each alignment file using the HaplotypeCaller function of the Genome Analysis Toolkit v4.1.8.1 [27]. We analyzed the frequency of occurrence (% of all reads of a pooled sample) of each SNV, insertions, and deletions (hits) using our in-house Python scripts, and compared them to the frequency of occurrence of the same hits in every other lineage. We considered mutations as spontaneously generated mutations only if no other lineages carried that variant in any depth and if hits reached at least 6% frequency of the reads at the corresponding position (theoretically, a spontaneously generated mutation in a pooled sample emerges with 20% or 16.7% frequency when 5 or 6 lineages are pooled, respectively, however, we allowed some variety when choosing 6% as a lower limit and 39.9% as an upper limit). Our analysis scripts for the full process are available through the following DOI: 10.6084/m9.figshare.25218191. Sequencing data are available at European Nucleotide Archive (ENA) under the PRJEB72651 project number. The umbrella project includes several datasets treated together (Table S4).

### 2.8. RNA Isolation, cDNA Synthesis and qPCR Experiments

Liquid cultures designated for qPCR analysis were cultivated in 100 mL volumes at 37 °C until reaching OD600 = 0.1 ± 0.02. Subsequently, a sublethal concentration of either BDQ, LZD, or CFZ (see Table 1) was added to half of the cultures, while the remaining half served as controls. The treatments were conducted for 8 h at 37 °C. Following treatment, the cultures were centrifuged (20 min, 3220× *g*, 4 °C) and the resulting pellets were utilized for RNA extraction.

RNA extraction was performed as follows. Cell pellets were resuspended in 2 mL RNA protect bacteria reagent (Qiagen; QIAGEN GmbH, Hilden, Germany, cat. no.:76506), incubated for 5 min at room temperature, and centrifuged for 20 min at 3220× *g* and at 4 °C before storage at −80 °C. Total RNA extraction was performed using the Qiagen RNeasy Mini kit (cat. no.: 74524). Cell disruption was performed in the manufacturer’s lysis buffer in the presence of glass beads during 5 × 1 min vortexing, followed by 1 min poses on ice. In the case of CFZ-treated samples, an extra step was also performed to get rid of the remaining CFZ from the lysate: 3 min of vortexing with equal volume of chloroform, followed by centrifugation (10 min, 3220× *g*; 4 °C). The aqueous phase was used for the next step. DNase digestion was performed in-column with Qiagen DNase I (cat. no.: 79254) for 90 min at room temperature. For quantitative and qualitative RNA analysis, we performed spectrometry using Nanodrop 2000 and non-denaturing 1% agarose gel electrophoresis (50 min/100 V), respectively. cDNA synthesis was carried out using the Applied Biosystems™ High-Capacity cDNA Reverse Transcription Kit with RNase Inhibitor (cat. no.: 4374967). A total quantity of 95–105 ng RNA was used for each reaction.

qPCR measurements were performed on a Bio-Rad CFX96 Touch™ Real-Time PCR Detection System. Primers were designed using IDT DNA oligo customizer (https://eu.idtdna.com/ (last accessed on 10 November 2024)), and were produced by Sigma Aldrich (for sequences, see Table S5). The qPCR reaction mixtures contained 7-7 nmoles of forward and reverse primers, 0.25 µL of the cDNA, Bioline Mytaq PCR premix (cat. no.: 25046), and VWR EvaGreen (cat. no.: #31000) in a total reaction volume of 10 µL. The thermal profile was the following: 95 °C/10 min, 50× (95 °C/10 s; 62 °C/10 s; 72 °C/10 s). Melting curves were registered between 55 °C and 95 °C, with an increment of 0.5 °C. The applied primers and their measured efficiencies are compiled in Table S5. The qPCR data were analyzed using the Bio-Rad CFX Maestro software v1.1 and numerically shown in Table S1. Non-reverse transcribed controls and no-template controls were used to account for any irrelevant DNA contamination. For all measurements, 3 technical and 3 biological replicates were used. All raw data can be found in 10.6084/m9.figshare.25218191. Changes in expression levels were determined in relation to the SigA (MSMEG_2758) and Ffh (MSMEG_2430) reference genes. We validated these references using GeNorm [28] analysis. SigA and Ffh proved to be stably expressed in our experimental system (Figure S2).

### 2.9. dNTP Extraction and Determination of the Cellular dNTP Concentrations

dNTP extraction and measurement were performed according to Szabo et al. [29]. Liquid cultures intended for dNTP analysis were grown in 100 mL volumes at 37 °C until they reached an OD600 of 0.1 ± 0.02. Subsequently, a sublethal concentration of antituberculotics (see Table 1) was added to half of the culture, while the remaining half served as a control. Following treatment (8 h; 37 °C), cultures were centrifuged (20 min, 3220× *g*, 4 °C) and the resulting pellets were resuspended in 0.5 mL of pre-cooled 60% methanol and kept at −20 °C overnight. After boiling for 5 min at 95 °C, the cell debris was removed by centrifugation (20 min, 13,400× *g*, 4 °C). The methanolic supernatant containing the soluble dNTP fraction was vacuum-dried with an Eppendorf Concentrator Plus device (Eppendorf, Hamburg, Germany) at 45 °C. Extracted dNTPs were dissolved in 50 μL nuclease-free water. In the case of CFZ treatment, the contaminating CFZ was extracted from the aqueous dNTP sample using an equal volume of chloroform (3 min vortex, then centrifugation at 3220× *g* for 10 min at 4 °C). Additionally, dGTP had to be retrieved from the organic phase in a further extraction step using equal volume of pH = 1 HCl solution (3 min vortex, than centrifugation). The aqueous phase was then used for dGTP measurement. Isolated dNTP samples were stored at −20 °C until used.

Determination of the dNTP pool size in each extract was performed in the following reaction mixture: 10 pmol template oligo (Sigma), 10 pmol probe (IDT) and 10 pmol NDP1 primer (Sigma) (see sequences in Table S6), 100 μM non-specific dNTP, 2.5 mM MgCl_2_, and 0.9 unit VWR^®^ TEMPase Hot Start DNA Polymerase (VWR) per 25 μL reaction. To record calibration curves, the reaction was supplied with 0–12 pmol specific dNTP. Fluorescence was recorded at every 13 s in a Bio-Rad CFX96 Touch™ Real-Time PCR Detection System or in a QuantStudio 1 qPCR instrument. The thermal profile was the following: 95 °C 15 min, (60 °C 13 s) × 260 cycle for dATP measurement, and 95 °C 15 min, (55 °C 13 s) × 260 cycle for dTTP, dCTP and dGTP measurements. Results were analyzed using the nucleoTIDY software v1.8 (http://nucleotidy.enzim.ttk.mta.hu/ last accessed on 10 November 2024) [29]. Results were given in molar concentrations for better comparison. To this end, cell volumes were calculated using ImageJ based on microscopic images of bacteria for every treatment. In addition to the graphical representation of the results, numerical data can be found in Table S3.

### 2.10. Statistics

We used an initial F-test to test the equality of variances of the tested groups. If the F-test hypothesis was accepted (*p* < 0.05), we used the two-way homoscedastic t-probe; if rejected, we used the two-way Welch’s t-probe to assess differences at a significance level *p* < 0.05 in each case. F- and t-statistics were counted for the ΔCt values [30] for the qPCR results and for the concentrations normalized to the cell volume in case of the dNTP measurements. We performed statistical analysis of the mutation rates by applying a *t*-test to the natural logarithm of the obtained mutation rate values.

## 3. Results

### 3.1. Adapting Stress Conditions and Assessing Their Impact on Cell Viability

To determine the potentially induced genome metabolic changes of commonly used second-line antituberculotics BDQ, LZD, and CFZ to mycobacteria, we optimized drug concentrations in liquid *M. smegmatis* culture and on plates as well, so that cells were affected by the drug but could be maintained through several generations. For the mutation accumulation studies, we used the drug concentrations optimized for plate-based experiments. However, for qPCR and dNTP measurements, we adjusted the drug concentrations in liquid cultures to ensure sufficient cell mass for further analysis (Table 1). As a positive control for direct DNA damage, ultraviolet (UV) irradiation was applied [31,32]. The optimization of the applied treatments is shown in Figure S1A,B.

### 3.2. Genomic Stability of M. smegmatis Under Antibiotic Pressure

For each stress treatment condition, we initiated and cultured 16 independent lineages of *M. smegmatis* mc^2^ 155, along with 56 lineages for the mock control, all originating from single colonies. Stress-treated lineages and a subset of mock lineages were cultivated on agar plates for 60 days. We calculated 6.3 ± 0.35 h of generation time on the plate in this experimental setup (for details, see Section 2). Thus, each lineage passed through ∼230 cell divisions. Some mock treatments were performed for 120 days to ensure a presumably sufficient number of mutational events without exposure to stress. Subsequent to treatment on solid plates, each lineage was expanded in a liquid culture devoid of drug pressure, and genomic DNA was isolated. 

Subsequently, all lineages were analyzed by WGS to identify mutational events induced by the drug treatments. We ensured a sequencing depth of at least 30–60× for all positions per independent lineage. The ancestor colony was also sequenced to identify pre-existing variations compared to the reference genome. According to the WGS results, our *M. smegmatis* ancestor strain harbored 151 different mutations compared to the *M. smegmatis* reference genome deposited in GenBank. These mutation events were identified in both treated and untreated lineages, and were excluded from further analysis as they represent specific variations of our laboratory strain. Additionally, we eliminated any mutations that appeared at the same position in any depth across other independent lineages.

**Figure 1:**
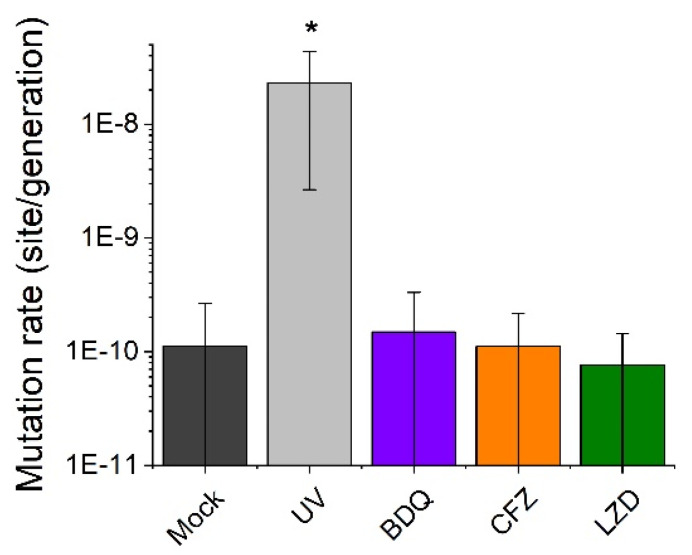
Mutation rates of wild-type *M. smegmatis* mc^2^ 155 strains under antibiotic pressure and DNA damaging stress (UV). Error bars represent standard deviations; * indicates that data are significantly different from the control (“Mock”) at the *p* = 0.05 level.

After a comprehensive analysis of the sequencing data, we observed an unexpectedly low incidence of newly generated mutations. Our findings indicated a maximum of 1 mutation per lineage during the 60-day drug treatments. Similarly, a maximum of 1 mutation event per lineage was identified during the 60- or 120-day mock treatment, resulting in 14 newly generated mutations for 56 lineages. Consequently, we computed an approximate mutation rate of 1.12 × 10^−10^ for the untreated *M. smegmatis* mc^2^ 155 strain. The mutation rates of all treated lineages fell within the same range (7–15 × 10^−11^), except for the UV treatment (2.4 × 10^−8^) employed as a positive control (Figure 1).

A subsequent analysis of each mutation, excluding those induced by UV treatment, was conducted to uncover potential adaptive changes. For BDQ, two out of five mutations impacted transporter proteins, and an additional two affected transcription factors (Table 2). In the case of LZD treatment, one out of two mutations affected a transporter protein, while in the case of CLF treatment, one of the three mutations affected a transcriptional regulator protein (Table 2). In the mock-treated group, among the 14 mutations, one impacted a transporter protein, and two affected transcription regulators. Most detected mutations (9 out of 14) impacted basic metabolic enzymes. Details regarding the genomic positions of mutations and their discernible consequences at the transcriptional or protein levels are provided in the supplementary archive associated with this article (10.6084/m9.figshare.25218191). None of the *de novo* generated mutations confer known adaptive variants.

**Figure 2:**
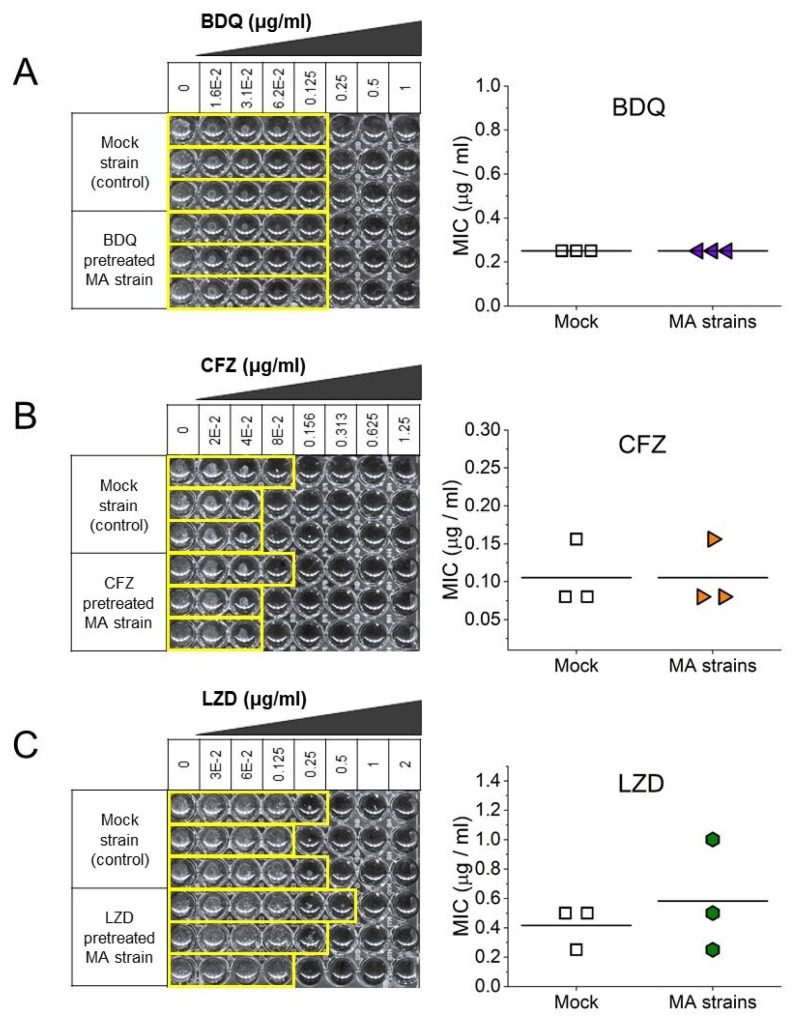
Sensitivity of *M. smegmatis* stress-treated and control (“Mock”) lines to the respective antibiotics (**A**) BDQ, (**B**) CFZ, and (**C**) LZD. Three randomly selected lines from both the mock-treated and stress-treated groups were resuscitated on plates containing the same stress conditions as those used in the MA experiment, and the MIC values were determined for the selected antibiotics.

Since we detected only a few mutations, we sought to determine whether our strains could develop tolerance to the applied treatment. The possible development of tolerance was evaluated by measuring the minimal inhibitory concentration (MIC) of both the mock-treated and stressed MA strains. In all cases, the MIC values showed no significant changes throughout the 60-day treatment (Figure 2).

### 3.3. Distinct Activation Patterns in the DNA Repair System

**Figure 3:**
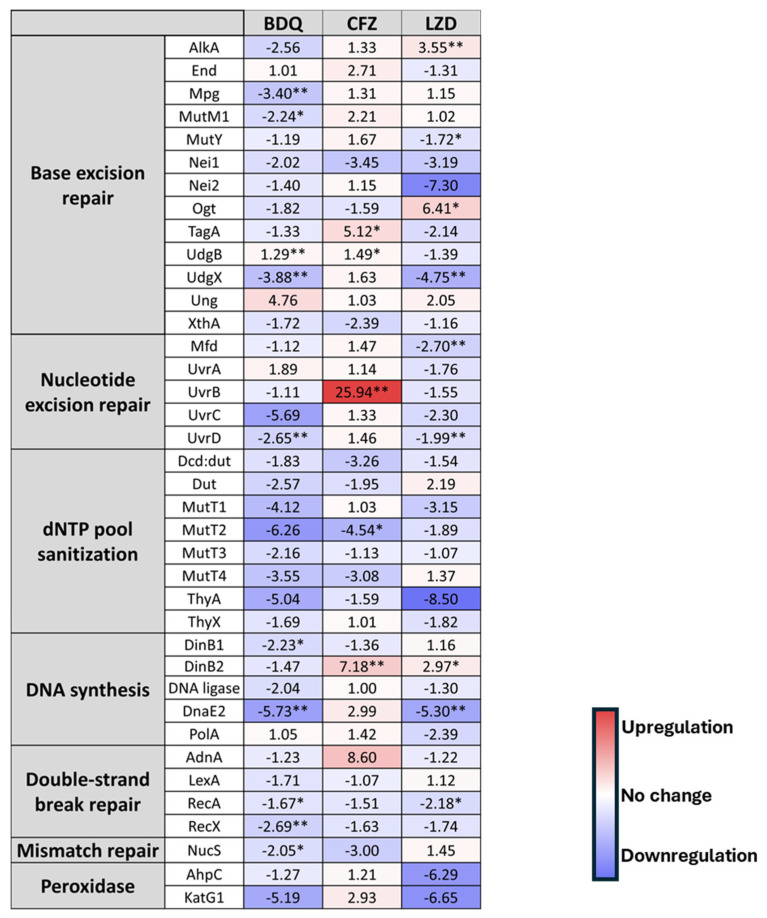
Changes in the expression of DNA repair genes upon stress treatments. Gene expression changes are normalized to the mock-treated control using the SigA and Ffh reference genes. Upregulation is interpreted as fold change; downregulation is interpreted as 1/(fold change). * *p* < 0.1; ** *p* < 0.05. The cells are color-coded, with upregulation indicated in red and downregulation in blue. The intensity of each color reflects the magnitude of change, and exact values are displayed numerically.

The mycobacterial DNA repair system is known to be highly robust, with several enzymes having redundant functions [33,34,35]. Although canonical mismatch repair proteins are not encoded in their genomes, NucS, a protein with a similar function, was described recently in mycobacteria [36]. In order to unveil potential mechanisms sustaining robust genome stability under the applied drug pressure, we explored the activation patterns of DNA repair pathways and other components of the stress response [37]. To this end, we subjected wild-type (wt) *M. smegmatis* to treatment with the second-line drugs and examined the expression patterns of all known DNA repair pathways at high precision using RT-qPCR. The measured relative expression levels are shown in Figure 3, and detailed information is provided in Table S1.

Upon BDQ treatment, we observed an overall downregulation pattern in DNA repair genes, except for some base excision repair (BER) enzymes (Figure 3). Conversely, CFZ, known for its DNA binding properties, activates the transcription of some DNA repair enzymes in each pathway. Notably, TagA and UdgB in BER, the UvrB helicase in nucleotide excision repair (NER), and the translesion polymerase DinB2 are particularly affected (Figure 3). Interestingly, at the same time, dNTP pool sanitation enzymes are downregulated, and the SOS response (including the LexA, RecA, and RecX enzymes) is not activated either (Figure 3). The LZD treatment results in selective AlkA, Ogt, and DinB2 activation. Conversely, other genes associated with the BER, NER, DNA double-strand break (DSB) repair, and dNTP pool sanitization are downregulated (Figure 3). 

### 3.4. dNTP Pool Reduction Is Induced by BDQ and CFZ

**Figure 4:**
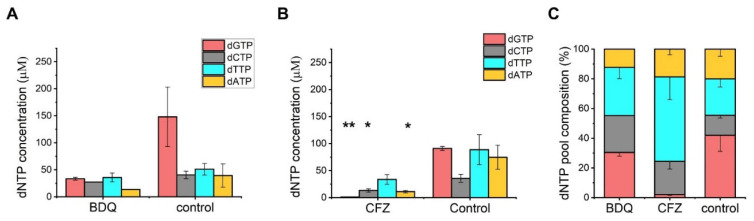
Treatment with antituberculotics BDQ and CFZ alters dNTP concentrations in the cell. (**A**) Cellular dNTP concentrations of BDQ-treated *M. smegmatis*. (**B**) Cellular dNTP concentrations of CFZ-treated *M. smegmatis*. dNTP quantities were measured in cellular extracts and normalized to the average cell volume to obtain the shown concentrations. Data bars represent the averages of three biological replicates, each carried out in three technical replicates. Error bars represent SE. The *p*-values of the *t*-tests calculated for the measured differences are: 0.12; ND; 0.29; 0.19 for dGTP; dCTP; dTTP; dATP for BDQ, and 0.02; 0.08; 0.18; 0.08 for dGTP; dCTP; dTTP; dATP in the case of CFZ, respectively. In the case of the BDQ treatment, two out of three biological parallels in the dCTP measurements were below the detection limit and, therefore, SE and significance could not be determined. ** and * indicate *p* < 0.05 and *p* < 0.1, respectively. (**C**) Relative composition of dNTP pools in drug-treated *M. smegmatis*.

We aimed to investigate whether BDQ and CFZ could alter the cellular dNTP pools given their known mechanism of action affecting nucleotide homeostasis [11,15], which, in turn, can influence mutagenesis. Therefore, we isolated dNTP from drug-treated cells and assessed cellular dNTP concentrations using a fluorescence-based detection method optimized in our laboratory [29]. Cellular concentrations were determined using cellular volumes derived from microscopy-based measurements of cellular dimensions (Table S2). Substantial changes in dNTP pools were measured following both BDQ and CFZ treatments (Figure 4 and detailed information in Table S3). Both treatments led to a decrease in the concentrations of all four dNTPs, with especially noticeable reductions seen in the amounts of purine nucleotides, dGTP, and dATP (Figure 4A,B). In the case of the CFZ treatment, dGTP levels decreased below the detection limit (Figure 4B). These changes considerably shift the usual cellular dNTP pool composition dominated by dGTP in *M. smegmatis* (Figure 4C).

## 4. Discussion

TB remains a medical challenge for several reasons, and one of them is the considerable genetic diversity revealed through the sequence analysis of *M. tuberculosis* isolates [38]. This diversity proves advantageous to the bacteria, leading to the emergence of variant subpopulations better equipped to exhibit improved resilience to stress. Importantly, this heterogeneity is also associated with an inherent resistance to first-line treatments. When choosing an antibiotic for DR-TB treatment, scientific criteria such as susceptibility patterns play a crucial role (unless the availability of the selected drug is limited). Additionally, it is desirable to know whether the chosen antibiotic has a mutation-promoting effect on the bacterial genome. Our research aimed primarily to gather information on the mutagenic potential and the basic molecular impacts of recently introduced second-line tuberculosis drugs on genome metabolism.

### 4.1. Second Line Antituberculotics Do Not Increase Mutation Rates in M. smegmatis

Previous literature reports have estimated mycobacterial mutation rates to be in the order of 10^−10^ per base pair per generation [39,40,41,42]. In our study, we focused on *M. smegmatis* to explore whether exposure to second-line antibiotics could elevate its mutation rates and, if so, elucidate the underlying molecular mechanisms. Using a mutation accumulation assay combined with whole genome sequencing, we searched for the emergence of drug-induced mutations in the genome. Remarkably, the mutation rate, estimated at around 10^−10^ base per generation, was not significantly increased by any of the second-line antibiotics BDQ, LZD, or CFZ in *M. smegmatis*. The only significant increase in mutation rate occurred upon UV treatment, which served as a positive control in our experiments (see Figure 1).

### 4.2. Mutations in Transporter Proteins and Transcription Regulators Are Overweighted in BDQ Treatment Without Known Adaptive Changes Detected

Following the assessment of each mutation under BDQ, CFZ, or LZD treatment, further analysis was carried out to identify potential adaptive changes. Intriguingly, for BDQ, two out of five mutations had an impact on transporter proteins (MSMEG_2927; MSMEG_4427), and an additional two affected transcription factors (MSMEG_0330; MSMEG_4025) (Table 2). In contrast, within the mock treatment group, the majority of mutations (9 out of 14) affected basic metabolic enzymes.

Specifically, MSMEG_0330 belongs to the LuxR-family regulators, which play crucial roles in the biosynthesis of secondary metabolites in actinobacteria. LuxR regulators are involved in various cellular processes such as quorum sensing, bioluminescence, virulence, and secondary metabolism [43]. MSMEG_4025 is a mycobacterial homolog of LysR-type transcriptional regulators, playing diverse roles in several cellular processes. These roles include the regulation of virulence, stress responses, motility, and amino acid metabolism [44,45]. MSMEG_2927 is a member of the OpuCB ABC transporter protein family in mycobacteria, fulfilling crucial functions associated with the response to osmotic stress and osmoregulation [46,47]. MSMEG_4427 is a homolog of the EmrB/QacA proteins, forming an integral part of the efflux system responsible for expelling antibiotics and various compounds from bacterial cells [48]. However, the identified mutation represents an early stop codon, indicating that it is unlikely to result in a functional efflux pump. 

Since none of the MIC values for the investigated second-line antibiotics increased significantly, it is unlikely that the observed mutations induced adaptive changes. Although there were initial signs that BDQ treatment led to a higher frequency of potential resistance-related mutations, further analysis found no evidence of adaptive changes or associated drug resistance.

### 4.3. Drugs Induce Specific Patterns in the DNA Repair Pathways

The intracellular nature of the TB pathogen necessitates its confrontation with various stress conditions and harmful agents, including reactive oxygen and nitrogen species within macrophages. Consequently, extensive genome-wide studies have examined stress-induced transcriptional changes in mycobacteria [49,50]. Specific activation of the DNA repair system in response to CIP [23,32] was reported. Despite *M. smegmatis* not being an intracellular pathogen, it shares DNA repair pathways with *M. tuberculosis* and is frequently used to model how mycobacteria handle DNA lesions [35]. Our investigation is focused on understanding stress-induced transcriptional changes in DNA repair pathways that could contribute to the preservation of genomic integrity under drug pressure.

BDQ, recently incorporated into DR-TB treatments inhibits the mycobacterial ATP synthase. The observed global reduction in the expression of DNA repair machinery components (Figure 2) correlates with a decrease in cellular energy levels. This comprehensive downregulation of the DNA repair apparatus implies a complete cessation of bacterial activity, potentially accounting for the observed minimal mutation events.

In contrast, CFZ, renowned for its affinity to DNA, triggers the increased transcription of several DNA repair enzymes. Our observations specifically indicate upregulation in BER and NER, as well as DSB repair pathways, playing crucial roles in preventing and repairing DNA lesions. The increased expression of these relevant DNA repair enzymes may contribute to sustaining a low mutation rate even under drug pressure. Notably, the error-prone polymerase DinB2 displayed a substantial upregulation without inducing a mutator phenotype. Simultaneously, reducing the dNTP pool may increase polymerase fidelity, ensuring proper DNA synthesis post-excision. This suggests that both error-prone and error-free repair mechanisms are concurrently activated, resulting in primarily error-free repairs. 

A shift in redox potential is a well-documented and common phenotypic stress response in mycobacteria [51]. While the precise mechanism of action of CFZ remains elusive, Yano et al. propose that the catalytic reduction of CFZ by NDH-2, followed by the spontaneous generation of ROS, might underlie its antibacterial effect [16]. In our study, ROS-induced cellular stress could be indirectly inferred from the expression of peroxidase genes *ahpC* and *katG1* [52]. However, CFZ treatment did not result in a significant change in the expression of these genes. Alternatively, the observed reduction in dGTP to undetectable levels is inherently bacteriostatic. Thus, it is possible that the NDH-2-specific reduction of CFZ is necessary because the reduced form of CFZ may play a role in dGTP depletion. 

Except for a few enzymes, LZD induced downregulation in BER, NER, DSB repair, DNA synthesis, and dNTP metabolism. Peroxidase enzymes also show a 6.3–6.7-fold reduction (at *p* < 0.14–0.18), indicating that ROS generation is not prevalent. Despite the general downregulation, there are some genes with increased expression. The prominently activated enzymes AlkA and Ogt, both glycosyl-transferases, play a role in the adaptive response against alkylation-induced DNA damage. The upregulation of enzymes involved in repairing alkylated or methylated DNA lesions suggests that these types of damage are prominent under LZD treatment conditions, while the downregulation of enzymes targeting other types of DNA damage implies a prioritization of repair pathways based on the types of lesions induced by the antibiotic. The induction of the error-prone polymerase DinB2 did not elicit a mutator phenotype, consistent with observations following CFZ treatment. The downregulation of DNA repair genes might be part of a broader cellular stress response, strategically directed at preserving energy and resources to ensure survival under adverse conditions. This regulatory mechanism allocates resources toward essential mechanisms crucial for the organism’s survival when facing antibiotic-induced stress [53,54].

### 4.4. BDQ and CFZ Decrease the Available Cellular dNTP

BDQ, as an ATPase synthesis inhibitor, has the potential to impact dNTP biosynthesis through the complex regulatory mechanism of the ribonucleotide reductase enzyme. Therefore, the observed reduction of the cellular dNTP pool, especially purine nucleotides, is consistent with its established mode of action. CFZ is known for its binding affinity to guanine bases in bacterial DNA, thereby inhibiting bacterial proliferation [55]. We hypothesized that CFZ might also bind to guanine bases within the dNTP pool, thus restricting the availability of dGTP for DNA biosynthesis. Our findings indeed showed that CFZ treatment led to a depletion of dGTP levels, likely hindering DNA synthesis and cellular replication. Whether this additional mechanism is connected to the oxidoreductive cycles previously identified for CFZ remains an open question.

## 5. Conclusions

Investigating the effects of second-line antituberculosis drugs on mycobacterial cellular homeostasis is of interest, especially in understanding their influence on genome integrity and the potential development of resistance through induced mutagenesis. Our study on *M. smegmatis* elucidated transcriptional changes in DNA repair systems and alterations in cellular dNTP levels upon BDQ, LZD, and CFZ treatments, providing additional insights into the molecular mechanisms of these antituberculosis drugs. Importantly, we demonstrated that the long-term treatment with BDQ, LZD, and CFZ did not induce mutator effects.

## Figures and Tables

**Table 1:** Overview of the administered drug treatments and their resulting effects on *M. smegmatis*. * controls shared with another study [23]. DSB: DNA double-strand break; N/A: not appliable; N/D: not determined.

Treatment				Liquid Culture Experiments		Agar Plate Experiments	
Category	Name	Abbreviation	Mechanism of Action	Subinhinitory Concentration [µg/mL]	CFU Compared to Control	Subinhinitory Concentration [µg/mL]	CFU Compared to Control
2nd line antibiotics	Bedaquiline	BDQ	ATP synthase inhibitor	0.5	51%	0.01	<0.1%
	Clofazimine	CFZ	Binds to DNA	5	30%	0.1	<0.1%
	Linezolide	LZD	Protein synthesis inhibitor	1	15%	0.1	12%
Positive Control	UV *		Pyr dimers, DSBs	ND	N/D	65	11%
None	Mock *		N/A	N/A	100%	N/A	100%

**Table 2:** Analysis of mutations detected upon treatment with second-line antituberculotics.

Position	Sample	Reference	Mutation	AA Mutation	Gene Code	Protein	Gene Ontology
366337	bed_B	G	GCCGGTACA	P217frameshift 221stop	MSMEG_0330	Transcriptional regulator, LuxR family protein	Regulation of transcription, DNA-dependent, Sequence-specific DNA binding, Phosphorelay response regulator activity, Phosphorelay signal transduction system, Intracellular
4098833	bed_B	T	C	Q29R	MSMEG_4025	Transcriptional regulator, LysR family protein	Sequence-specific DNA binding transcription factor activity, Regulation of transcription, DNA-dependent
193256	bed_C	C	T	N/A	N/A	N/A	Intergenic region
2990225	bed_C	C	T	G165D	MSMEG_2927	ABC transporter, permease protein OpuCB	Transport, Plasma membrane, Transporter activity, Integral to membrane
4508029	bed_C	G	A	W167stop	MSMEG_4427	Transmembrane efflux pump	Transmembrane transport, Integral to membrane
2006423	clf_A	T	TG	M138frameshift	MSMEG_1928	protein-serine/threonine phosphatase	Phosphoprotein phosphatase activity
2784157	clf_C	C	T	A37T	MSMEG_2713	Peptidase M52, hydrogen uptake protein	Peptidase activity, Enzyme activator activity
5994006	clf_C	G	A	T301T	MSMEG_5933	ArsR family transcriptional regulator	Regulation of transcription, DNA-dependent, DNA binding
971780	lzd_A	A	G	V344A	MSMEG_0886	serine/threonine-protein kinase PknD	Protein phosphorylation, Protein serine/threonine kinase activity, ATP binding
5293841	lzd_B	T	TGGGCGCCGCTATTGCGGCCGC	V378frameshift	MSMEG_5194	Integral membrane protein	Transmembrane transport

## Data Availability

The data underlying this article are available in the Figshare repository, DOI: 10.6084/m9.figshare.25218191. Sequencing data are available at European Nucleotide Archive (ENA) with PRJEB72651 project number under an umbrella project.

## Supplementary Materials

The following supporting information can be downloaded at: https://www.mdpi.com/article/10.3390/biom14111451/s1, Figure S1: optimization of drug treatments; Table S1: average normalized gene expressions; Table S2: cellular parameters of treated cells; Table S3: dNTP measurements; Table S4: WGS samples deposited at ENA webserver; Table S5: primer sequences and efficiencies used for qPCR; Figure S2: stability analysis of reference genes; Table S6: primer sequences for dNTP measurements. The data underlying this article are available in the Figshare repository, DOI: 10.6084/m9.figshare.25218191. Sequencing data are available from the European Nucleotide Archive (ENA) with project number PRJEB72651 under an umbrella project.

## References

[1] Doucet-Populaire F., Buriankova K., Weiser J., Pernodet J.-L. (2002). Natural and acquired macrolide resistance in mycobacteria. Curr. Drug Targets Infect. Disord..

[2] Gygli S.M., Borrell S., Trauner A., Gagneux S. (2017). Antimicrobial resistance in Mycobacterium tuberculosis: Mechanistic and evolutionary perspectives. FEMS Microbiol. Rev..

[3] Dookie N., Rambaran S., Padayatchi N., Mahomed S., Naidoo K. (2018). Evolution of drug resistance in Mycobacterium tuberculosis: A review on the hmolecular determinants of resistance and implications for personalized care. J. Antimicrob. Chemother..

[4] Zhang Z.-Y., Sun Z.-Q., Wang Z.-L., Hu H.-R., Wen Z.-L., Song Y.-Z., Zhao J.-W., Wang H.-H., Guo X.-K., Zhang S.-L. (2013). Identification and pathogenicity analysis of a novel non-tuberculous mycobacterium clinical isolate with nine-antibiotic resistance. Clin. Microbiol. Infect..

[5] Verbenko D.A., Solomka V.S., Kozlova I.V., Kubanov A.A. (2021). The genetic determinants of *Mycobacterium leprae* resistance to antimicrobial drugs. Vestn. Dermatol. I Venerol..

[6] Global Tuberculosis Report 2023. https://www.who.int/teams/global-tuberculosis-programme/tb-reports/global-tuberculosis-report-2023.

[7] Matteelli A., Carvalho A.C., E Dooley K., Kritski A. (2010). TMC207: The first compound of a new class of potent anti-tuberculosis drugs. Futur. Microbiol..

[8] Mahajan R. (2013). Bedaquiline: First FDA-approved tuberculosis drug in 40 years. Int. J. Appl. Basic Med. Res..

[9] Padmapriyadarsini C., Vohra V., Bhatnagar A., Solanki R., Sridhar R., Anande L., Muthuvijaylakshmi M., Rana M.B., Jeyadeepa B., Taneja G. (2022). Bedaquiline, Delamanid, Linezolid and Clofazimine for Treatment of Pre-extensively Drug-Resistant Tuberculosis. Clin. Infect. Dis..

[10] Gopal M., Padayatchi N., Metcalfe J.Z., O’Donnell M.R. (2013). Systematic review of clofazimine for the treatment of drug-resistant tuberculosis. Int. J. Tuberc. Lung Dis..

[11] Sarathy J.P., Gruber G., Dick T. (2019). Re-Understanding the Mechanisms of Action of the Anti-Mycobacterial Drug Bedaquiline. Antibiotics.

[12] Wang Z., Soni V., Marriner G., Kaneko T., Boshoff H.I.M., Barry C.E., Rhee K.Y. (2019). Mode-of-action profiling reveals glutamine synthetase as a collateral metabolic vulnerability of M. tuberculosis to bedaquiline. Proc. Natl. Acad. Sci. USA.

[13] Hashemian S.M., Farhadi T., Ganjparvar M. (2018). Linezolid: A review of its properties, function, and use in critical care. Drug Des. Dev. Ther..

[14] Stadler J.A.M., Maartens G., Meintjes G., Wasserman S. (2023). Clofazimine for the treatment of tuberculosis. Front. Pharmacol..

[15] Morrison N.E., Marley G.M. (1976). Clofazimine binding studies with deoxyribonucleic acid. Int. J. Lepr. Other Mycobact. Dis..

[16] Yano T., Kassovska-Bratinova S., Teh J.S., Winkler J., Sullivan K., Isaacs A., Schechter N.M., Rubin H. (2011). Reduction of clofazimine by mycobacterial type 2 NADH:quinone oxidoreductase: A pathway for the generation of bactericidal levels of reactive oxygen species. J. Biol. Chem..

[17] Oliva B., O’neill A.J., Miller K., Stubbings W., Chopra I. (2004). Anti-staphylococcal activity and mode of action of clofazimine. J. Antimicrob. Chemother..

[18] Mothiba M.T., Anderson R., Fourie B., Germishuizen W.A., Cholo M.C. (2015). Effects of clofazimine on planktonic and biofilm growth of Mycobacterium tuberculosis and Mycobacterium smegmatis. J. Glob. Antimicrob. Resist..

[19] Sparks I.L., Derbyshire K.M., Jacobs W.R., Morita Y.S. (2023). Mycobacterium smegmatis: The Vanguard of Mycobacterial Research. J. Bacteriol..

[20] Kurthkoti K., Varshney U. (2012). Distinct mechanisms of DNA repair in mycobacteria and their implications in attenuation of the pathogen growth. Mech. Ageing Dev..

[21] Houghton J., Townsend C., Williams A.R., Rodgers A., Rand L., Walker K.B., Böttger E.C., Springer B., Davis E.O. (2012). Important role for Mycobacterium tuberculosis UvrD1 in pathogenesis and persistence apart from its function in nucleotide excision repair. J. Bacteriol..

[22] Mohan A., Padiadpu J., Baloni P., Chandra N. (2015). Complete Genome Sequences of a Mycobacterium smegmatis Laboratory Strain (MC2 155) and Isoniazid-Resistant (4XR1/R2) Mutant Strains. Genome Announc..

[23] Molnár D., Surányi É.V., Trombitás T., Füzesi D., Hirmondó R., Tóth J. (2024). Genetic Stability of *Mycobacterium smegmatisunder* the Stress of First-Line Antitubercular Agents: Assessing Mutagenic Potential. bioRxiv.

[24] Bolger A.M., Lohse M., Usadel B. (2014). Trimmomatic: A flexible trimmer for Illumina sequence data. Bioinformatics.

[25] Langmead B., Salzberg S.L. (2012). Fast gapped-read alignment with Bowtie 2. Nat. Methods.

[26] Faust G.G., Hall I.M. (2014). SAMBLASTER: Fast duplicate marking and structural variant read extraction. Bioinformatics.

[27] McKenna A., Hanna M., Banks E., Sivachenko A., Cibulskis K., Kernytsky A., Garimella K., Altshuler D., Gabriel S., Daly M. (2010). The Genome Analysis Toolkit: A MapReduce framework for analyzing next-generation DNA sequencing data. Genome Res..

[28] Sundaram V.K., Sampathkumar N.K., Massaad C., Grenier J. (2019). Optimal use of statistical methods to validate reference gene stability in longitudinal studies. PLoS ONE.

[29] Szabó J.E., Surányi V., Mébold B.S., Trombitás T., Cserepes M., Tóth J. (2020). A user-friendly, high-throughput tool for the precise fluorescent quantification of deoxyribonucleoside triphosphates from biological samples. Nucleic Acids Res..

[30] Yuan J.S., Reed A., Chen F., Stewart C.N. (2006). Statistical analysis of real-time PCR data. BMC Bioinform..

[31] Crowley D.J., Boubriak I., Berquist B.R., Clark M., Richard E., Sullivan L., DasSarma S., McCready S. (2006). The uvrA, uvrB and uvrC genes are required for repair of ultraviolet light induced DNA photoproducts in *Halobacterium* sp. NRC-1. Saline Syst..

[32] O’Sullivan D.M., Hinds J., Butcher P.D., Gillespie S.H., McHugh T.D. (2008). Mycobacterium tuberculosis DNA repair in response to subinhibitory concentrations of ciprofloxacin. J. Antimicrob. Chemother..

[33] Srinath T., Bharti S.K., Varshney U. (2007). Substrate specificities and functional characterization of a thermo-tolerant uracil DNA glycosylase (UdgB) from Mycobacterium tuberculosis. DNA Repair.

[34] Kurthkoti K., Srinath T., Kumar P., Malshetty V.S., Sang P.B., Jain R., Manjunath R., Varshney U. (2010). A distinct physiological role of MutY in mutation prevention in mycobacteria. Microbiology.

[35] Singh A. (2017). Guardians of the mycobacterial genome: A review on DNA repair systems in Mycobacterium tuberculosis. Microbiology.

[36] Castañeda-García A., Prieto A.I., Rodríguez-Beltrán J., Alonso N., Cantillon D., Costas C., Pérez-Lago L., Zegeye E.D., Herranz M., Plociński P. (2017). A non-canonical mismatch repair pathway in prokaryotes. Nat. Commun..

[37] Romero D., Traxler M.F., López D., Kolter R. (2011). Antibiotics as signal molecules. Chem. Rev..

[38] Sun G., Luo T., Yang C., Dong X., Li J., Zhu Y., Zheng H., Tian W., Wang S., Barry C.E. (2012). Dynamic population changes in Mycobacterium tuberculosis during acquisition and fixation of drug resistance in patients. J. Infect. Dis..

[39] Ragheb M.N., Ford C.B., Chase M.R., Lin P.L., Flynn J.L., Fortune S.M. (2013). The mutation rate of mycobacterial repetitive unit loci in strains of M. tuberculosis from cynomolgus macaque infection. BMC Genom..

[40] Kucukyildirim S., Long H., Sung W., Miller S.F., Doak T.G., Lynch M. (2016). The Rate and Spectrum of Spontaneous Mutations in Mycobacterium smegmatis, a Bacterium Naturally Devoid of the Postreplicative Mismatch Repair Pathway. G3 Genes Genomes Genet..

[41] Ford C.B., Lin P.L., Chase M.R., Shah R.R., Iartchouk O., Galagan J., Mohaideen N., Ioerger T.R., Sacchettini J.C., Lipsitch M. (2011). Use of whole genome sequencing to estimate the mutation rate of Mycobacterium tuberculosis during latent infection. Nat. Genet..

[42] Colangeli R., Arcus V.L., Cursons R.T., Ruthe A., Karalus N., Coley K., Manning S.D., Kim S., Marchiano E., Alland D. (2014). Whole genome sequencing of Mycobacterium tuberculosis reveals slow growth and low mutation rates during latent infections in humans. PLoS ONE.

[43] Li Z., Li X., Xia H. (2022). Roles of LuxR-family regulators in the biosynthesis of secondary metabolites in Actinobacteria. World J. Microbiol. Biotechnol..

[44] Zhang L., Yu S., Ning X., Fang H., Li J., Zhi F., Li J., Zhou D., Wang A., Jin Y. (2022). A lysr transcriptional regulator manipulates macrophage autophagy flux during brucella infection. Front. Cell. Infect. Microbiol..

[45] Modrzejewska M., Kawalek A., Bartosik A.A. (2021). The LysR-Type Transcriptional Regulator BsrA (PA2121) Controls Vital Metabolic Pathways in *Pseudomonas aeruginosa*. Msystems.

[46] Kappes R.M., Kempf B., Kneip S., Boch J., Gade J., Meier-Wagner J., Bremer E. (1999). Two evolutionarily closely related ABC transporters mediate the uptake of choline for synthesis of the osmoprotectant glycine betaine in Bacillus subtilis. Mol. Microbiol..

[47] Kappes R.M., Kempf B., Bremer E. (1996). Three transport systems for the osmoprotectant glycine betaine operate in Bacillus subtilis: Characterization of OpuD. J. Bacteriol..

[48] De Rossi E., Aínsa J.A., Riccardi G. (2006). Role of mycobacterial efflux transporters in drug resistance: An unresolved question. FEMS Microbiol. Rev..

[49] Briffotaux J., Liu S., Gicquel B. (2019). Genome-Wide Transcriptional Responses of Mycobacterium to Antibiotics. Front. Microbiol..

[50] Li W., Sanchez-Hidalgo A., Jones V., de Moura V.C.N., North E.J., Jackson M. (2017). Synergistic Interactions of MmpL3 Inhibitors with Antitubercular Compounds In Vitro. Antimicrob. Agents Chemother..

[51] Niki M., Niki M., Tateishi Y., Ozeki Y., Kirikae T., Lewin A., Inoue Y., Matsumoto M., Dahl J.L., Ogura H. (2012). A novel mechanism of growth phase-dependent tolerance to isoniazid in mycobacteria. J. Biol. Chem..

[52] Wayne L.G., Diaz G.A. (1986). A double staining method for differentiating between two classes of mycobacterial catalase in polyacrylamide electrophoresis gels. Anal. Biochem..

[53] Arrigoni R., Ballini A., Topi S., Bottalico L., Jirillo E., Santacroce L. (2022). Antibiotic Resistance to Mycobacterium tuberculosis and Potential Use of Natural and Biological Products as Alternative Anti-Mycobacterial Agents. Antibiotics.

[54] Gupta K.R., Arora G., Mattoo A., Sajid A. (2021). Stringent response in mycobacteria: From biology to therapeutic potential. Pathogens.

[55] Arbiser J.L., Moschella S.L. (1995). Clofazimine: A review of its medical uses and mechanisms of action. J. Am. Acad. Dermatol..

