# Supplementary material for: Assessing the Impact of Bedaquiline, Clofazimine, and Linezolid on Mycobacterial Genome Integrity
Source: Biomolecules. 2024 Nov 15;14(11):1451. doi: 10.3390/biom14111451 (PMC11591709; doi:10.3390/biom14111451)
Supplement: Supplementary file 1 [file biomolecules-14-01451-s001.zip › biomolecules-3256633-supplementary.pdf]

Fig S1 Optimization of drug treatments. Viability of wt *M. smegmatis* in liquid cultures (A) and on plates (B) containing second line antituberculotics. Relative viability is calculated upon CFU after 8 hours of drug treatment or CFU of exponentially growing wt *M. smegmatis* cultures on plates containing the respective antibiotic at varying concentrations.

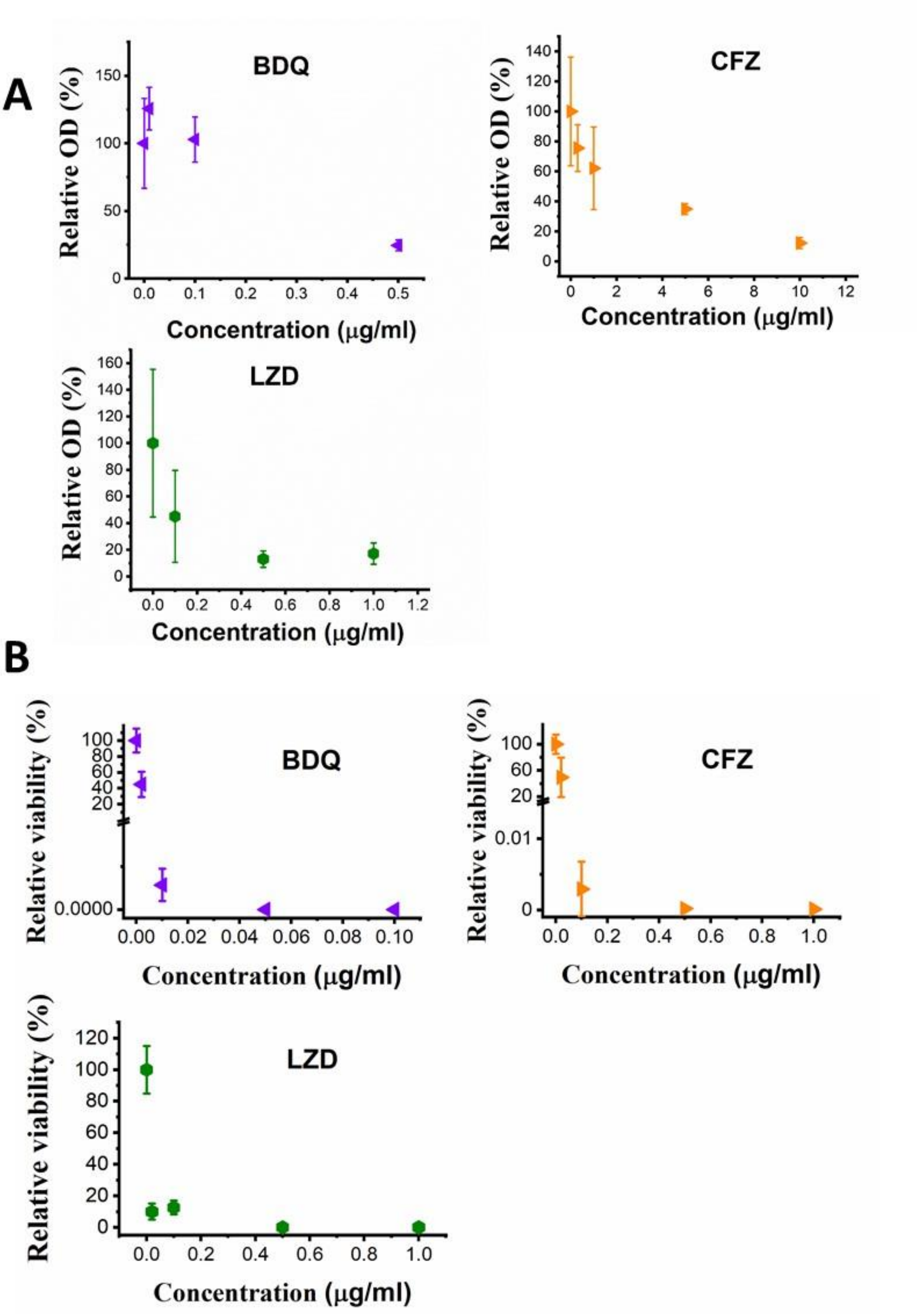

Table S1: Average normalized gene expressions. SEM= standard error of the mean. For raw data, see 10.6084/m9.figshare.25218191

REFERENCE				SigA/Ffh
**Target**	**Treatment**	**Fold change**	**+/- SEM**	**p**
AdnA	CFZ	8.60	0.42	0.19
	NT	1.00	0.05	
AhpC	CFZ	1.21	0.10	0.77
	NT	1.00	0.10	
AlkA	CFZ	1.33	0.20	0.82
	NT	1.00	0.17	
Dcd:dut	CFZ	0.31	0.02	0.26
	NT	1.00	0.91	
DinB1	CFZ	0.74	0.11	0.59
	NT	1.00	0.54	
DinB2	CFZ	7.18	0.66	0.00
	NT	1.00	0.05	
DNA ligase	CFZ	1.00	0.06	0.97
	NT	1.00	0.12	
DnaE2	CFZ	2.99	0.38	0.12
	NT	1.00	0.08	
Dut	CFZ	0.51	0.04	0.73
	NT	1.00	0.48	
End	CFZ	2.71	0.17	0.22
	NT	1.00	0.09	
KatG1	CFZ	2.93	0.21	0.17
	NT	1.00	0.10	
LexA	CFZ	0.93	0.08	0.97
	NT	1.00	0.13	
Mfd	CFZ	1.47	0.15	0.38
	NT	1.00	0.13	
Mpg	CFZ	1.31	0.23	0.13
	NT	1.00	0.36	
MutM1	CFZ	2.21	0.13	0.49
	NT	1.00	0.06	
MutT1	CFZ	1.03	0.09	0.81
	NT	1.00	0.14	
MutT2	CFZ	0.22	0.03	0.07
	NT	1.00	0.60	
MutT3	CFZ	0.88	0.27	0.96
	NT	1.00	0.44	
MutT4	CFZ	0.32	0.02	0.20
	NT	1.00	0.46	
MutY	CFZ	1.67	0.13	0.80
	NT	1.00	0.26	
Nei1	CFZ	0.29	0.06	0.38

	**NT**	**1.00**	**1.14**	
Nei2	**CFZ**	**1.15**	**0.16**	**0.87**
	**NT**	**1.00**	**0.24**	
NucS	**CFZ**	**0.33**	**0.03**	**0.59**
	**NT**	**1.00**	**0.80**	
Ogt	**CFZ**	**0.63**	**0.07**	**0.45**
	**NT**	**1.00**	**0.40**	
PolA	**CFZ**	**1.42**	**0.10**	**0.72**
	**NT**	**1.00**	**0.24**	
RecA	**CFZ**	**0.66**	**0.04**	**0.36**
	**NT**	**1.00**	**0.23**	
RecX	**CFZ**	**0.61**	**0.05**	**0.80**
	**NT**	**1.00**	**0.50**	
TagA	**CFZ**	**5.12**	**0.45**	**0.07**
	**NT**	**1.00**	**0.06**	
ThyA	**CFZ**	**0.63**	**0.04**	**0.67**
	**NT**	**1.00**	**0.21**	
ThyX	**CFZ**	**1.01**	**0.09**	**0.95**
	**NT**	**1.00**	**0.10**	
UdgB	**CFZ**	**1.49**	**0.15**	**0.10**
	**NT**	**1.00**	**0.21**	
UdgX	**CFZ**	**1.63**	**0.11**	**0.78**
	**NT**	**1.00**	**0.15**	
Ung	**CFZ**	**1.03**	**0.19**	**0.93**
	**NT**	**1.00**	**0.35**	
UvrA	**CFZ**	**1.14**	**0.13**	**0.76**
	**NT**	**1.00**	**0.12**	
UvrB	**CFZ**	**25.94**	**1.40**	**0.01**
	**NT**	**1.00**	**0.05**	
UvrC	**CFZ**	**1.33**	**0.19**	**0.77**
	**NT**	**1.00**	**0.23**	
UvrD	**CFZ**	**1.46**	**0.09**	**0.39**
	**NT**	**1.00**	**0.08**	
XthA	**CFZ**	**0.42**	**0.05**	**0.24**
	**NT**	**1.00**	**0.63**	
AdnA	**BDQ**	**0.82**	**0.17**	**0.59**
	**NT**	**1.00**	**0.23**	
AhpC	**BDQ**	**0.79**	**0.03**	**0.83**
	**NT**	**1.00**	**0.14**	
AlkA	**BDQ**	**0.39**	**0.05**	**0.21**
	**NT**	**1.00**	**0.22**	
Dcd:dut	**BDQ**	**0.55**	**0.03**	**0.58**
	**NT**	**1.00**	**0.22**	
DinB1	**BDQ**	**0.45**	**0.18**	**0.08**
	**NT**	**1.00**	**1.13**	
DinB2	**BDQ**	**0.68**	**0.04**	**0.31**
	**NT**	**1.00**	**0.53**	

DNA ligase	**BDQ**	**0.49**	**0.03**	**0.11**
	**NT**	**1.00**	**0.13**	
DnaE2	**BDQ**	**0.17**	**0.02**	**0.00**
	**NT**	**1.00**	**2.28**	
Dut	**BDQ**	**0.39**	**0.03**	**0.10**
	**NT**	**1.00**	**0.45**	
End	**BDQ**	**1.01**	**0.07**	**0.99**
	**NT**	**1.00**	**0.11**	
KatG1	**BDQ**	**0.19**	**0.02**	**0.27**
	**NT**	**1.00**	**0.16**	
LexA	**BDQ**	**0.58**	**0.05**	**0.17**
	**NT**	**1.00**	**0.53**	
Mfd	**BDQ**	**0.89**	**0.07**	**0.86**
	**NT**	**1.00**	**0.25**	
Mpg	**BDQ**	**0.29**	**0.05**	**0.01**
	**NT**	**1.00**	**1.03**	
MutM1	**BDQ**	**0.45**	**0.03**	**0.07**
	**NT**	**1.00**	**0.35**	
MutT1	**BDQ**	**0.24**	**0.02**	**0.11**
	**NT**	**1.00**	**0.20**	
MutT2	**BDQ**	**0.16**	**0.01**	**0.26**
	**NT**	**1.00**	**1.79**	
MutT3	**BDQ**	**0.46**	**0.02**	**0.33**
	**NT**	**1.00**	**0.29**	
MutT4	**BDQ**	**0.28**	**0.02**	**0.41**
	**NT**	**1.00**	**0.33**	
MutY	**BDQ**	**0.84**	**0.38**	**0.86**
	**NT**	**1.00**	**0.70**	
Nei1	**BDQ**	**0.50**	**0.09**	**0.58**
	**NT**	**1.00**	**0.34**	
Nei2	**BDQ**	**0.72**	**0.06**	**0.73**
	**NT**	**1.00**	**0.13**	
NucS	**BDQ**	**0.49**	**0.10**	**0.09**
	**NT**	**1.00**	**0.60**	
Ogt	**BDQ**	**0.55**	**0.04**	**0.59**
	**NT**	**1.00**	**0.21**	
PolA	**BDQ**	**1.05**	**0.06**	**0.96**
	**NT**	**1.00**	**0.10**	
RecA	**BDQ**	**0.60**	**0.03**	**0.08**
	**NT**	**1.00**	**0.16**	
RecX	**BDQ**	**0.37**	**0.02**	**0.04**
	**NT**	**1.00**	**0.47**	
TagA	**BDQ**	**0.75**	**0.09**	**0.63**
	**NT**	**1.00**	**0.23**	
ThyA	**BDQ**	**0.20**	**0.01**	**0.22**

	NT	1.00	0.47	
ThyX	BDQ	0.59	0.03	0.64
	NT	1.00	0.14	
UdgB	BDQ	1.29	0.05	0.03
	NT	1.00	0.25	
UdgX	BDQ	0.26	0.03	0.01
	NT	1.00	0.15	
Ung	BDQ	4.76	2.50	0.29
	NT	1.00	0.06	
UvrA	BDQ	1.89	0.11	0.47
	NT	1.00	0.09	
UvrB	BDQ	0.90	0.05	0.82
	NT	1.00	NA	
UvrC	BDQ	0.18	0.02	0.17
	NT	1.00	0.29	
UvrD	BDQ	0.38	0.03	0.03
	NT	1.00	0.19	
XthA	BDQ	0.58	0.04	0.59
	NT	1.00	0.42	
AdnA	LZD	0.82	0.07	0.28
	NT	1.00	0.11	
AhpC	LZD	0.16	0.00	0.18
	NT	1.00	0.24	
AlkA	LZD	3.55	0.25	0.05
	NT	1.00	0.05	
Dcd:dut	LZD	0.65	0.05	0.61
	NT	1.00	0.10	
DinB1	LZD	1.16	0.22	0.88
	NT	1.00	0.24	
DinB2	LZD	2.97	0.28	0.08
	NT	1.00	0.05	
DNA ligase	LZD	0.77	0.03	0.36
	NT	1.00	0.19	
DnaE2	LZD	0.19	0.02	0.03
	NT	1.00	0.39	
Dut	LZD	2.19	0.11	0.29
	NT	1.00	0.04	
End	LZD	0.77	0.04	0.40
	NT	1.00	0.11	
KatG1	LZD	0.15	0.01	0.14
	NT	1.00	0.81	
LexA	LZD	1.12	0.13	0.85
	NT	1.00	0.08	
Mfd	LZD	0.37	0.03	0.04
	NT	1.00	0.12	
Mpg	LZD	1.15	0.11	0.81
	NT	1.00	0.13	

MutM1	**LZD**	**1.02**	**0.04**	**0.78**
	**NT**	**1.00**	**0.05**	
MutT1	**LZD**	**0.32**	**0.02**	**0.17**
	**NT**	**1.00**	**0.11**	
MutT2	**LZD**	**0.53**	**0.03**	**0.47**
	**NT**	**1.00**	**0.17**	
MutT3	**LZD**	**0.93**	**0.06**	**0.92**
	**NT**	**1.00**	**0.08**	
MutT4	**LZD**	**1.37**	**0.14**	**0.69**
	**NT**	**1.00**	**0.05**	
MutY	**LZD**	**0.58**	**0.05**	**0.08**
	**NT**	**1.00**	**0.27**	
Nei1	**LZD**	**0.31**	**0.03**	**0.30**
	**NT**	**1.00**	**0.20**	
Nei2	**LZD**	**0.14**	**0.01**	**0.20**
	**NT**	**1.00**	**0.42**	
NucS	**LZD**	**1.45**	**0.11**	**0.53**
	**NT**	**1.00**	**0.08**	
Ogt	**LZD**	**6.41**	**0.32**	**0.08**
	**NT**	**1.00**	**0.05**	
PolA	**LZD**	**0.42**	**0.01**	**0.11**
	**NT**	**1.00**	**0.13**	
RecA	**LZD**	**0.46**	**0.03**	**0.09**
	**NT**	**1.00**	**0.07**	
RecX	**LZD**	**0.57**	**0.03**	**0.50**
	**NT**	**1.00**	**0.15**	
TagA	**LZD**	**0.47**	**0.02**	**0.35**
	**NT**	**1.00**	**0.14**	
ThyA	**LZD**	**0.12**	**0.01**	**0.12**
	**NT**	**1.00**	**0.43**	
ThyX	**LZD**	**0.55**	**0.03**	**0.60**
	**NT**	**1.00**	**0.08**	
UdgB	**LZD**	**0.72**	**0.06**	**0.78**
	**NT**	**1.00**	**0.12**	
UdgX	**LZD**	**0.21**	**0.01**	**0.01**
	**NT**	**1.00**	**0.12**	
Ung	**LZD**	**2.05**	**0.14**	**0.58**
	**NT**	**1.00**	**0.04**	
UvrA	**LZD**	**0.57**	**0.03**	**0.37**
	**NT**	**1.00**	**0.06**	
UvrB	**LZD**	**0.64**	**0.03**	**0.49**
	**NT**	**1.00**	**0.12**	
UvrC	**LZD**	**0.44**	**0.03**	**0.29**
	**NT**	**1.00**	**0.13**	
UvrD	**LZD**	**0.50**	**0.02**	**0.02**
	**NT**	**1.00**	**0.10**	
XthA	**LZD**	**0.86**	**0.06**	**0.50**

	**NT**	**1.00**	**0.09**	

Table S2: Cellular parameters of BDQ and CFZ treated *M. smegmatis* cells analyzed by microscopic measurements

Strain	N	Cell length (µm)	SD	With (µm)	SD	Cell volume (µm3)
BDQ	117	3.78	1.26	1.13	0.11	3.80
control	96	6.36	2.46	0.84	0.20	3.56
CFZ	100	3.90	1.23	1.10	0.10	3.71
control	116	6.32	2.01	0.90	0.08	4.02

Table S3: Results and statistical analysis of dNTP measurements

**Treatment**	**dNTP**	**Sample**	**uM**	**+/-SEM**	**Fold change**	**+/- SE**	**p**
**BDQ**	dGTP	Treated	33.45	2.70	0.23	0.02	0.12
		Non-treated	147.96	55.06			
	dCTP	Treated	27.33	nd	0.67	nd	nd
		Non-treated	40.50	7.13			
	dTTP	Treated	35.72	8.30	0.70	0.16	0.29
		Non-treated	51.13	10.81			
	dATP	Treated	13.57	nd	0.35	0.25	0.19
		Non-treated	39.33	21.46			
**CFZ**	dGTP	Treated	1.21	0.25	0.01	0.00	0.02
		Non-treated	91.13	3.55			
	dCTP	Treated	13.28	3.08	0.37	0.09	0.08
		Non-treated	35.75	7.50			
	dTTP	Treated	33.69	9.07	0.38	0.10	0.18
		Non-treated	88.79	27.66			
	dATP	Treated	11.11	2.48	0.15	0.03	0.08
		Non-treated	74.68	22.24			

Table S4: WGS samples deposited at ENA webserver, with PRJEB72651 project number under an umbrella project

Treatment	Sample	Sample description	ENA filename	Experiment length
Clofazimine	CLFA	5 different lineages of clofazimine treated *Mycobacterium smegmatis*	CLFA.rg.bam	60 days
	CLFB	5 different lineages of clofazimine treated *Mycobacterium smegmatis*	CLFB.rg.bam	60 days
	CLFC	6 different lineages of clofazimine treated *Mycobacterium smegmatis*	CLFC.rg.bam	60 days
Bedaquiline	BedA	5 different lineages of bedaquiline treated *Mycobacterium smegmatis*	BedA.rg.bam	60 days
	BedB	6 different lineages of bedaquiline treated *Mycobacterium smegmatis*	BedB.rg.bam	60 days
	BedC	5 different lineages of bedaquiline treated *Mycobacterium smegmatis*	BedC.rg.bam	60 days
	BedD	5 different lineages of bedaquiline treated *Mycobacterium smegmatis*	BedD.rg.bam	60 days
Linezolid	LzdA	5 different lineages of linezolid treated *Mycobacterium smegmatis*	LzdA.rg.bam	60 days
	LzdB	6 different lineages of linezolid treated *Mycobacterium smegmatis*	LzdB.rg.bam	60 days
	LzdC	5 different lineages of linezolid treated *Mycobacterium smegmatis*	LzdC.rg.bam	60 days

Table S5 Primer sequences and primer efficiencies used for qPCR

**Target**	**Msm Gene ID**	**Sequence (5’□3’)**	**Efficiency (%)**
**AdnA_for**	**MSMEG_1941**	CGCAGTCCTACCGTTGC	**81.6**
**AdnA_rev**		CTTCGGCGTGTGTGGAG	
**AhpC_for**	**MSMEG_4753**	CCGACAAGCCCGAGAAG	**103**
**AlhpC_rev**		GGAACGTGGACCGGATG	
**AlkA_for**	**MSMEG_4925**	GCCTCCATCCGTCAGTTC	**83.5**
**AlkaA_rev**		GCCGAACAATCCCTCGTAG	
**Dcd:dut_for**	**MSMEG_0678**	CGGTTGGAGGGCAAGTC	**98.2**
**Dcd:dut_rev**		CACAGCTGCCCGATCTTC	
**DinB1_for**	**MSMEG_3172**	TCACGGTCAAGCTCAAGAAG	**83.2**
**DinB1_rev**		AACCCAACGCCCACAAG	
**DinB2_for**	**MSMEG_6443**	CCAGTTACGAGGCCAAGG	**81.2**
**DinB2_rev**		GGGTGGTGTCGTGGAAG	
**DNA ligase_for**	**MSMEG_2362**	GGAGGTCAAACGCAAGGG	**91.1**
**DNA ligase_rev**		CAACTCGGTTCCGCACTC	
**DnaE2_for**	**MSMEG_1633**	GCCTCGCTGGTGTTCTAC	**87.7**
**DnaE2_rev**		AGGCTGGCATTCACGTC	
**Dut_for**	**MSMEG_2765**	ATACCGCACGGAATGGTC	**97.1**
**Dut_rev**		GTCTGCGGATCCAGGTTG	
**End_for**	**MSMEG_1383**	AGCGCATCAAGTCCATCAC	**83**

**End_rev**		GCCGTCTCGCAGATCAC	
**Ffh_for**	**MSMEG_2430**	GAGCTCATCGGCATCCTC	**86.2**
**Ffh_rev**		GGGCTGTGACCCTTGTC	
**KatG1_for**	**MSMEG_6384**	GCCCATCGGAGAAGCTC	**96**
**KatG1_rev**		CCTCGTCCAGCGGATTG	
**LexA_for**	**MSMEG_2740**	GTTCCTGCTCAAGGTCGTC	**105**
**LexA_rev**		CATGAGCCACACCTGACC	
**Mfd_for**	**MSMEG_5423**	GAGCTCACCCGGTTCAC	**89**
**Mfd_rev**		CTTGGTGGCGGTCTTCTC	
**Mpg_for**	**MSMEG_3759**	GTGCGGAACTCGGTGATG	**89.6**
**Mpg_rev**		GCTGTGCACTGTCGACTC	
**MutM1_for**	**MSMEG_2419**	CAGGCGCGGAAAGTACC	**93**
**MutM1_rev**		CGCTGATCGACGAAGCTC	
**MutT1_for**	**MSMEG_2390**	GGTGGACAAGCTCGTATGG	**107**
**MutT1_rev**		GCGATCGTCACCCTTGTAG	
**MutT2_for**	**MSMEG_5148**	GGCTGTGGGAACTTCCTG	**86.7**
**MutT2_rev**		TGTCATGGCGTCGTTGAG	
**MutT3_for**	**MSMEG_0790**	CGTACACGACGGTGATCG	**92.1**
**MutT3_rev**		GTAGGCGCTGCCAACTC	
**MutT4_for**	**MSMEG_6927**	CAGGCGGTCTGGTCATC	**87.1**
**MutT4_rev**		TGGATCCCGGTCTCCTC	
**MutY_for**	**MSMEG_6083**	GGGAAAGCTCGGCTACC	**86.4**
**MutY_rev**		GGAACGCTCGCCTGATAG	
**Nei1_for**	**MSMEG_4683**	CATCGGCGCTCAGTACG	**99.7**
**Nei1_rev**		CGGTCTGCGTGACTTGG	
**Nei2_for**	**MSMEG_1756**	GCGGTACCGACATGGAC	**92.1**
**Nei2_rev**		GAAACACAACTCGTTGCAGTAG	
**NucS_for**	**MSMEG_4923**	CGCGCTACCTGGAACTG	**99.7**
**NucS_rev**		GTACTCGTCGCTGTCCATTC	
**Ogt_for**	**MSMEG_4928**	AGATCCCGTACGGACAGAC	**92.6**
**Ogt_rev**		CCCATAACCCGTGAGACTTC	
**PolA_for**	**MSMEG_3839**	GAGCTCACCCGGTTCAC	**83.9**
**PolA_rev**		CTTGGTGGCGGTCTTCTC	
**ProC_for**	**MSMEG_0943**	GCCCGGCGTACTTCTTC	**73.3**
**ProC_rev**		CGGCGTTCACCTGATCC	
**RecA_for**	**MSMEG_2723**	CGCGTCAAGGTCGTCAAG	**90.1**
**RecA_rev**		ACCCTCGTAGGTGAACCAG	
**RecX_for**	**MSMEG_2724**	CTCGAAACCCAGCTGACC	**89.9**
**RecX_rev**		GAGCTCGACAGCCAAGG	
**SigA_for**	**MSMEG_2758**	CATCTGCTGGAGGCGAAC	**87.7**
**SigA_rev**		CTTGTAGCCCTTGGTGTAGTC	
**TagA_for**	**MSMEG_5082**	GACTACCACGACACCGAATG	**91.4**
**TagA_rev**		GGATCGAACCCGTGGAAC	
**ThyA_for**	**MSMEG_2670**	GTCGGGTGAGCACATCG	**92.8**
**ThyA_rev**		GGCGACGTAGAACTGGAAG	
**ThyX_for**	**MSMEG_2683**	CGTACAGCTGATCGCCAAG	**94.2**
**ThyX_rev**		CGTTGGTCGCGGTCTTC	

**UdgB_for**	**MSMEG_5031**	ACGTTGACCACCGCATAC	**97.7**
**UdgB_rev**		TTCGTTCCCGATCTGCTTG	
**UdgX_for**	**MSMEG_0265**	CCCGGTGACAAAGAGGAC	**91.8**
**UdgX_rev**		CTTGTGGATGCGTCGTTTG	
**Ung_for**	**MSMEG_2399**	CCGTGGCAGATCAGGTG	**89.2**
**Ung_rev**		TGTCGGGTAGGGATCCTG	
**UvrA_for**	**MSMEG_3808**	CCTGGGCATCCGCAAAG	**94.6**
**UvrA_rev**		CCCATCTCCTCACCGAGAC	
**UvrB_for**	**MSMEG_3816**	GAGAAGGACAGCTCGATCAAC	**96.6**
**UvrB_rev**		CTGACCCACCTGCAACTC	
**UvrC_for**	**MSMEG_3078**	TCGATTTCTGCGACTTCCTG	**88.1**
**UvrC_rev**		CCACGGCCTGTTTCTCC	
**UvrD_for**	**MSMEG_5534**	GGGAGGACGGCATGTTC	**86.3**
**UvrD_rev**		CGCGATTCCGGGTTGAG	
**XthA_for**	**MSMEG_0829**	CCTACCACGGGCTCAAC	**80.7**
**XthA_rev**		GCACGTAGAGGCTCCATAC	

Fig S2: geNorm analysis based reference gene stability used in the qPCR measurements outlined in the methods section.

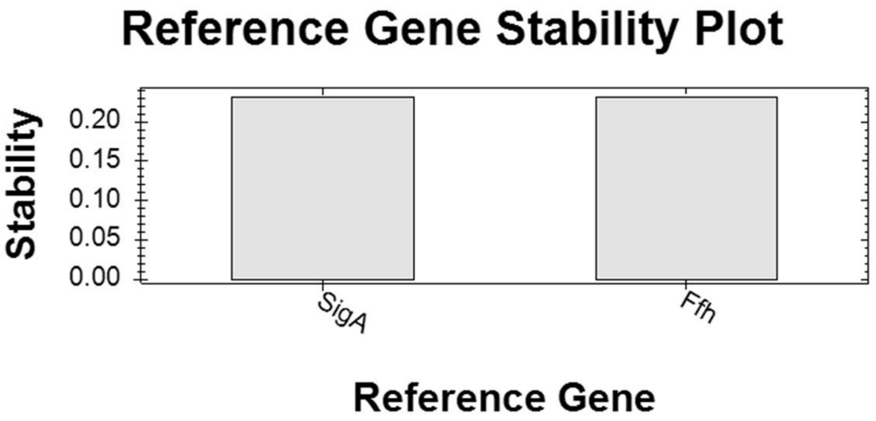

Table S6: Primer sequences for dNTP measurements

Name	Sequence (5'→3')
NDP-1 primer	CCGCCTCCACCGCC
FAM-dTTP probe	**6-FAM/**AGGACCGAG**/ZEN/**GCAAGAGCGAGCGA**/IBFQ**
FAM-dATP probe	**6-FAM/**TGGTCCGTG**/ZEN/**GCTTGTGCGTGCGT**/IBFQ**
FAM-dGTP probe	**6-FAM/**ACCATTCAC**/ZEN/**CTCACACTCACTCC**/IBFQ**
FAM-dCTP probe	**6-FAM/**AGGATTGAG**/ZEN/**GTAAGAGTGAGTGG**/IBFQ**
dTTP-DT1 template	TCGCTCGCTCTTGCCTCGGTCCTTT**A**TTTGGCGGTGGAGGCGG
dATP-DT1 template	ACGCACGCACAAGCCACGGACCAAA**T**AAAGGCGGTGGAGGCGG
dCTP-DT1 template	CCACTCACTCTTACCTCAATCCTTT**G**TTTGGCGGTGGAGGCGG
dGTP-DT2 template	GGAGTGAGTGTGAGGTGAATGGTTT**C**TTT**C**TTTGGCGGTGGAGGCGG
